# Prolonged in situ self-healing in structural composites via thermo-reversible entanglement

**DOI:** 10.1038/s41467-022-33936-z

**Published:** 2022-10-31

**Authors:** Alexander D. Snyder, Zachary J. Phillips, Jack S. Turicek, Charles E. Diesendruck, Kalyana B. Nakshatrala, Jason F. Patrick

**Affiliations:** 1 Department of Mechanical and Aerospace Engineering, North Carolina State University (NCSU), 1840 Entrepreneur Dr., Raleigh, NC 27695 USA; 2 Department of Civil, Construction, and Environmental Engineering, North Carolina State University, 915 Partners Way, Raleigh, NC 27695 USA; 3 Schulich Faculty of Chemistry, Technion-Israel Institute of Technology, Haifa, 3200003 Israel; 4 Department of Civil and Environmental Engineering, University of Houston (UH), 4726 Calhoun Rd., Houston, TX 77204 USA

**Keywords:** Composites, Aerospace engineering, Mechanical engineering, Mechanical properties

## Abstract

Natural processes continuously degrade a material’s performance throughout its life cycle. An emerging class of synthetic self-healing polymers and composites possess property-retaining functions with the promise of longer lifetimes. But sustained in-service repair of structural fiber-reinforced composites remains unfulfilled due to material heterogeneity and thermodynamic barriers in commonly cross-linked polymer-matrix constituents. Overcoming these inherent challenges for mechanical self-recovery is vital to extend in-service operation and attain widespread adoption of such bioinspired structural materials. Here we transcend existing obstacles and report a fiber-composite capable of minute-scale and prolonged in situ healing — 100 cycles: an order of magnitude higher than prior studies. By 3D printing a mendable thermoplastic onto woven glass/carbon fiber reinforcement and co-laminating with electrically resistive heater interlayers, we achieve in situ thermal remending of internal delamination via dynamic bond re-association. Full fracture recovery occurs below the glass-transition temperature of the thermoset epoxy-matrix composite, thus preserving stiffness during and after repair. A discovery of chemically driven improvement in thermal remending of glass- over carbon-fiber composites is also revealed. The marked lifetime extension offered by this self-healing strategy mitigates costly maintenance, facilitates repair of difficult-to-access structures (e.g., wind-turbine blades), and reduces part replacement, thereby benefiting economy and environment.

## Abstract (web summary)

Synthetic materials that can repeatedly self-repair, akin to biological systems, are vital to meeting the 21st century’s infrastructural demands. Here, authors develop fiber-reinforced composites with rapid and prolonged in situ self-healing while also preserving structural integrity.

## Introduction

The ability to heal and recover from minor injuries is vital for living organisms^1^. The success of biological healing stems from two essential attributes: (i) In situ: an innate capacity to deliver healing agents to the injured site and self-repair the damage in place. (ii) Sustained: the healing functionality persists even after many damage/repair cycles (i.e., throughout an organism’s life). For instance, upon laceration in healthy human skin, healing begins instantly and continues until restoring necrotic with new tissue—without hindering other body functionalities^2^. Bereft of in situ and sustained repair, a benign cut could exacerbate and prove fatal.

Traditional synthetic material systems, on the other hand, lack the ability to self-repair. Consequently, they are often over-engineered to prevent failure. However, such a design paradigm is not always practical nor achievable: conservative designs can lead to bulky structures, and structural systems invariably encounter unexpected conditions over a long operating lifetime. Fiber-reinforced polymer (FRP) composites—proven transformative in the aerospace industry—are modern materials replacing conventional constituents (e.g., metals) for superior strength- and stiffness-to-weight ratios and enhanced durability in corrosive environments. However, such hierarchical FRP composites are prone to complex, multi-scale damage modes^3,4^ (e.g., matrix cracking and interlaminar delamination) that are difficult to detect using current nondestructive evaluation (NDE) or structural health monitoring (SHM) techniques^5,6^. These defects at the micron-scale can develop into large-scale damage and, if unaddressed, may lead to catastrophic failures.

Even upon locating internal damage, manual repair of FRP composites is costly, laborious, and not always successful^7^. Moreover, the widespread replacement of FRP is not sustainable from environmental and economical standpoints. The dwindling supply of non-renewable natural resources, adverse climatic effects from production-related pollution^8^, difficulty in recycling spent thermoset-matrix materials^9^, and already pervasive plastic pollution^10^ make a strong environmental case for finding alternatives to FRP component replacement^11^. Coupled with high energy demands from manufacturing^12^ and growing supply chain shortages for petrochemicals used to produce polymer products^13^, the need to extend the useful lifetime of FRP materials is now greater than ever. It is, therefore, prudent to embrace and realize synthetic structural systems that mimic living organisms’ evolutionary healing attributes of in situ and continual self-repair.

Over the last twenty years, self-healing polymers and composites have emerged, offering an autonomous route to mitigate deterioration and prolong useful lifetime^14–17^. However, in situ self-healing FRP composites—that can recover repeatedly from structural-scale damage—have yet to transcend research laboratories^18–20^. In recent years, the industry has adopted self-healing coatings that rely on embedded microcapsules filled with reactive liquid chemistries; upon a surface scratch, the liquid healing agents—released from the ruptured capsule core—polymerize to restore the protective coating^21^. But small volumes of healing agents within microcapsules limit the size of damage that is repairable, and the inability to replenish payload precludes multiple heal cycles. Microvascular systems, which leverage nature’s solution to nutrient transport in living organisms^22,23^ (e.g., blood circulation), have overcome capsule damage volume limitations^24^ and can achieve multiple in situ self-healing cycles of internal fracture in structural polymers and composites^22,25,26^. However, despite recent techniques to create complex vascular networks in laminated composites^27,28^, several fundamental issues persist: inadequate mixing of liquid chemistries^26^, accumulation of healed polymer debris, and cross-contamination of healing agents blocking vasculature^18^. Such standing issues limit the number of repeat repair cycles and impede translation to real-world applications^29^.

Contrary to the extrinsic capsule- and vascular-based self-healing, intrinsic repair strategies rely on dynamic bond reassociation within the host material^18,19,21,23^. While markedly successful in soft materials systems (e.g., gels, elastomers)^30–33^, intrinsic healing in structural polymers is inherently difficult and requires specialized chemistries (e.g., covalent adaptable networks) or external energy input (e.g., heat) to overcome kinetic barriers for segmental dynamics, bond activation and reassociation^15,17,20,34–36^. Intrinsic healing via thermal remending occurs primarily above the glass-transition temperature (*T*_*g*_) at which the rigid polymer becomes rubbery with diminished elastic modulus, precluding in situ self-repair in a structural setting. Moreover, commercially prevalent thermoset fiber-composite matrices (i.e., epoxies) do not include reversible chemical cross-links even above *T*_*g*_. On the other hand, thermoplastics readily undergo polymer chain re-entanglement beyond their melting point (*T*_*m*_). Poly(ethylene-co-methacrylic acid) (EMAA)—a commodity copolymer thermoplastic—has been shown to exhibit self-healing in neat films^37^ and also deployed in various forms (e.g., particles, fibers) within FRP composites to restore mechanical performance via thermal remending^38–42^. However, to date, researchers have only realized FRP repair either ex situ (i.e., in an oven) or above *T*_*g*_ of the composite matrix; the former eliminates the ability to achieve in-service repair, while the latter compromises structural function during healing and elicits irreversible changes. Importantly, recovery often diminishes with repeated healing, and the number of consecutive heal cycles in such material systems has not surpassed ten^38^, which coincidentally matches the highest cycle count reported for vascular self-healing in fiber-composites^43^. Thus, despite the wealth of research over the past two decades, sustained in situ self-healing within synthetic FRP composites has remained elusive.

**Fig. 1:**
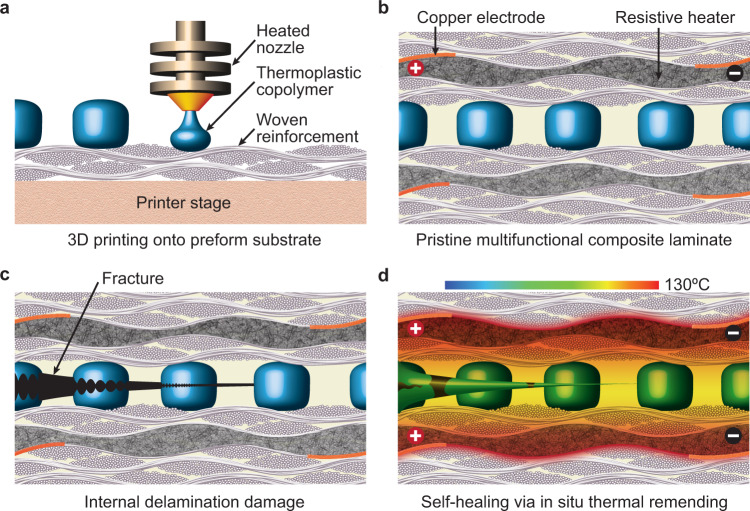
Overview concept of self-healing via in situ thermal remending. **a** 3D printed micro-domains of polyethylene-co-methacrylic acid (EMAA) directly onto woven fiber-reinforcement. **b** Fiber-composite laminate constructed by infusion of the epoxy matrix into a symmetrically-stacked textile preform containing EMAA-modified reinforcement and resistive heater interlayers. **c** Upon interlaminar delamination in the composite, cohesive fracture ruptures EMAA as opposed to adhesive separation from composite—due to strong interfacial bonding between the EMAA and woven reinforcement. **d** Electrical power input to resistive heaters raises the composite temperature to 130 ^∘^C—which is higher than the melting point of EMAA (*T*_*m*_ ≈80 ^∘^C), but lower than the glass-transition temperature of the epoxy matrix (*T*_*g*_ ≈150 ^∘^C)—thus preserving the structural integrity of the composite during in situ repair. EMAA domains melt and flow into contact where chemical bond reassociation (i.e., polymer chain re-entanglement) heals the cohesively damaged interfaces repeatedly via thermal remending.

In this article, we demonstrate prolonged, in situ self-healing in an FRP composite without compromising structural integrity during repair via thermal remending (Fig. 1). Our approach relies on three critical attributes: (i) precision patterning of EMAA micro-domains and ensuring interfacial bonding to woven fiber-reinforcement, (ii) achieving in situ heat generation to melt the thermoplastic copolymer, and (iii) EMAA chain re-entanglement occurring below *T*_*g*_ of the thermoset epoxy matrix to retain elastic modulus during the repair. We find that substantial and sustained self-healing is achieved primarily via thermo-reversible hydrogen bonding between fractured EMAA interfaces, where micro-topology evolution and covalent surface interactions differentiate the healing behavior of glass- versus carbon-fiber composites.

## Results and discussion

### Self-healing composite fabrication

3D printing via fused-deposition modeling (FDM) is employed to pattern molten EMAA directly onto woven textile reinforcement, forming a strong adhesive bond to the micro-textured fibrous surface (Fig. 1a). Upon printing, a layer of reinforcement is placed atop the EMAA-patterned substrate and subsequently heated to melt-bond the upper textile layer (see Methods Section). Two resistive heaters, similar in thickness to the primary reinforcing plies (≈250 μm), are incorporated into a symmetric stacking sequence and co-laminated by vacuum-assisted infusion of an aerospace-grade epoxy matrix (Fig. 1b). A key design feature for achieving repeated repair: by melt-bonding EMAA domains directly to fiber-reinforcement before epoxy matrix incorporation, we promote a cohesive fracture instead of an adhesive separation from the matrix/reinforcement (Fig. 1c). Electrical power input to the integrated resistive heaters melts the cohesively fractured EMAA domains, whereby direct contact of the molten thermoplastic interfaces achieves repeated thermal remending via dynamic bond reassociation (Fig. 1d).

**Fig. 2:**
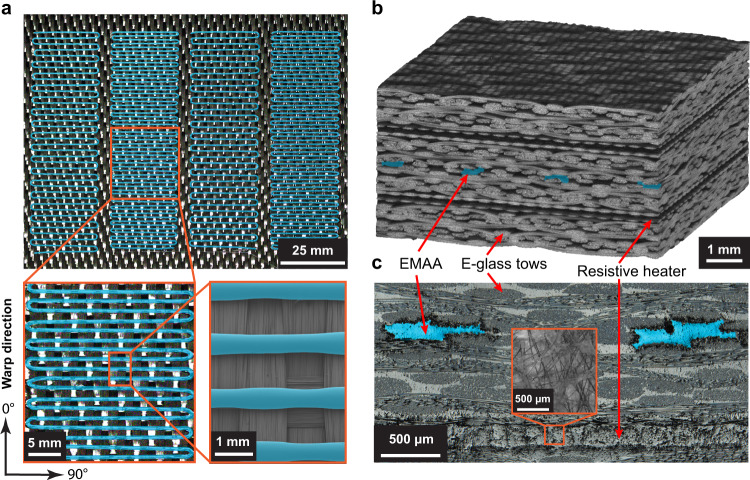
Hierarchical multifunctional composite construction. **a** Overhead images of 3D printed serpentine patterns of EMAA thermoplastic (blue overlay) directly onto woven carbon-fiber reinforcement demonstrating both scalable (top) and precise (bottom) placement. **b** 3D x-ray computed microtomography (*μ*CT) reconstruction of self-healing glass-fiber-reinforced composite. **c** Cross-section of laminated composite revealing EMAA melt-bonded to micro-fiber bundles (tows) and resistive heater interlayers containing a percolating network of electrically conductive carbon-whiskers (inset).

Prolonged self-healing via thermo-mechanical multifunctionality is enabled by heterogeneous material hierarchy. High-resolution (μm) and scalable (mm – m) 3D printing of EMAA serpentine micro-patterns (300 × 500 μm) is carried out directly on 2D textile reinforcement. The conformable structural fabric is woven from glass/carbon fiber-bundles (i.e., tows) containing thousands of individual fibers (5–10 μm diameter), which ensures a strong interfacial bond via high surface area contact with the printed EMAA (Fig. 2a). The melt-bonded layers along with additional reinforcing plies and two resistive heater interlayers are co-laminated in a symmetric stacking sequence as verified by X-ray computed microtomography of a cured composite (Fig. 2b). This symmetric architecture mitigates warping upon thermoset epoxy matrix solidification and undesirable coupling among stress states (e.g., axial-shear). A heated post-cure furthers matrix cross-linking and enhances the mechanical integrity—strength, stiffness, and toughness—as well as thermal stability (*T*_*g*_ ≈140 ^∘^C) of the FRP composite. The embedded resistive heaters, comprising a percolating network of conductive carbon-whiskers (≈10 μm diameter) suspended within an adhesive layer between dielectric glass-fiber (Fig. 2c), provide a confined electrical current pathway for efficient (≈99%) conversion of electrical to thermal energy via in situ Joule heating. The multifunctional fiber-reinforced composite largely exhibits elastic structural behavior, although cohesively fractured EMAA domains exhibit ductile tearing. This predominately elastic response ensures registration and intimate contact of molten thermoplastic even after delamination, enabling repeated re-bonding of interfaces via polymer chain entanglement.

### Thermo-mechanical characterization

**Fig. 3:**
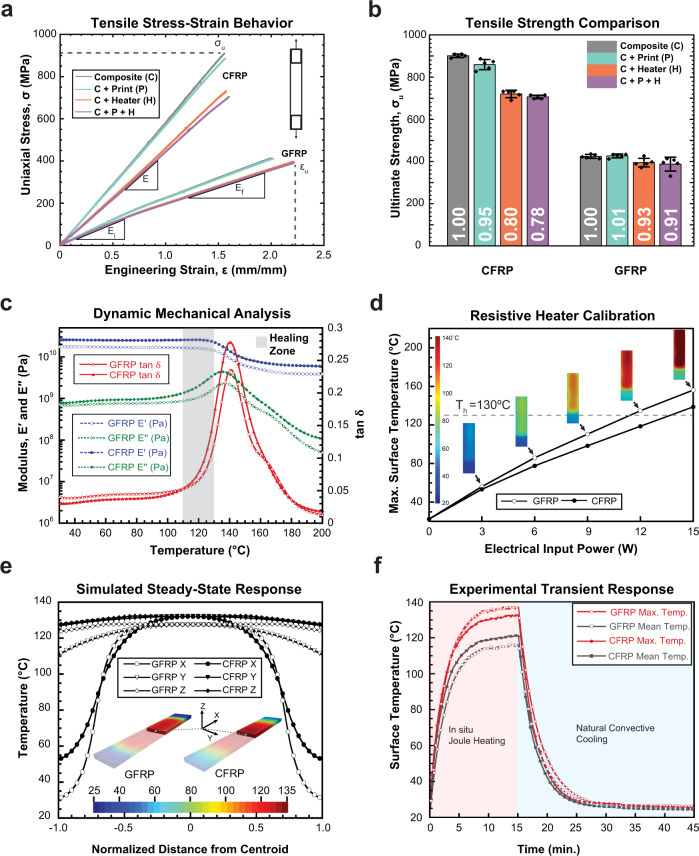
Thermo-mechanical material characterization. **a** Uniaxial tensile response for various glass- (GFRP) and carbon-fiber (CFRP) composite laminates, including plain and permuted configurations with printed EMAA mid-layer and resistive heaters. **b** Failure strength summary of composite laminates in (**a**); numbers at the bottom of the bar plots indicate the normalized strength values in reference to plain composite controls. Error bars represent the standard deviation from the mean (*n* = 5). **c** Dynamic mechanical analysis (DMA) behavior for GFRP and CFRP composites with matching glass-transition temperatures ≈140 ^∘^C. **d** Resistive heater electrical power input versus thermal output calibration curve including top surface temperature contours via infrared (IR) imaging of glass- and carbon-fiber composite specimens. **e** Steady-state temperature profiles for GFRP and CFRP composites via 3D finite element simulations at 12 and 13.5 W input power, respectively. **f** Transient experimental in situ heating and cooling behavior for GFRP and CFRP laminates over a 45 min thermal remending cycle (15 min heating followed by 30 min cooling).

For translating our self-healing system to real-world applications, the material-level modifications above must preserve structural performance in comparison to unaltered composites. To confirm this preservation of performance, three microstructural variations for glass-fiber (GFRP) and carbon-fiber-reinforced polymer (CFRP) composites were studied: (1) laminates with EMAA printed on the central reinforcement layer, (2) laminates with two resistive heaters replacing respective fiber-reinforcement layers, and (3) laminates containing both resistive heaters and a printed mid-layer of EMAA. All laminated composite variations (i.e., plain along with modified architectures) exhibit either a linear (in CFRP) or bilinear (in GFRP) tensile response (Fig. 3a). Both glass- and carbon-fiber composites containing only the EMAA mid-layer (type 1) show a relatively minor (<10%) reduction in ultimate tensile strength (*σ*_*u*_) (Fig. 3b). Meanwhile, composites with the resistive heater interlayers (type 2) exhibit differing strength reductions depending on the primary reinforcing material. In GFRP, since the resistive heaters themselves are comprised of adhesively bonded woven glass layers, the strength reduction is statistically no different than a plain composite control. However, in CFRP, which is inherently stronger (>2x *σ*_*u*_) than GFRP, an anticipated reduction in strength from resistive heater ply substitutions is roughly 25%. In both GFRP and CFRP, the strength and modulus reductions are additive: composites containing heater and EMAA interlayers (type 3) are nearly equivalent to combining the individual (type 1 and 2) tensile effects.

To ascertain mechanical properties are maintained during repair—essential for a truly in situ strategy—dynamic mechanical analysis (DMA) is undertaken to characterize the elastic modulus at elevated temperatures (up to 200 ^∘^C) for both glass- and carbon-fiber composites (Fig. 3c). The glass-transition temperature (*T*_*g*_), marked by the peak of tan *δ*, is ≈140 ^∘^C, beyond which the elastic storage modulus (${E}^{\prime}$) decreases significantly. At 10 ^∘^C above *T*_*g*_, only 36 and 35% of the elastic modulus at room temperature (RT) are retained in glass- and carbon-fiber composites, respectively. However, at 10 ^∘^C below *T*_*g*_ (i.e., the target healing temperature), 84 and 86% of the respective elastic moduli at RT for glass- and carbon-fiber composites are retained (SI Sec. 1). While staying below *T*_*g*_ of the thermoset-matrix is critical to preserve composite stiffness, maintaining a sufficient remending temperature is important to ensure low flow resistance (i.e., viscosity) of the molten thermoplastic healing agent so as to achieve adequate distribution within confined cracks. In this regard, one advantage of EMAA is its low viscosity compared to other commodities printable thermoplastics^44,45^; at the target healing temperature (*T*_*h*_ = 130 ^∘^C), the melt-flow viscosity of EMAA is 440 Pa ⋅ s (SI Sec. 2): only twice that at *T*_*g*_. To achieve the target healing temperature via Joule heating, roughly 12 W of electrical power is required (i.e., 1/5th of a 60 W light bulb) for a glass-fiber composite, where a linear relationship between input power and maximum surface temperature is observed in both glass and carbon composites via infrared (IR) imaging (Fig. 3d). Since the IR camera can only monitor surface temperature, we resorted to numerical modeling for ensuring the temperature in the interior remains below *T*_*g*_ at the target input power(s) for GFRP/CFRP composites. Fig. 3e shows the results from a full 3D finite element heat transfer simulation, accounting for anisotropic thermal conductivity (Fourier model) within the body, natural convective (Newton’s law of cooling) and nonlinear radiative (Stefan-Boltzmann law) heat transfer on the boundaries (SI Sec. 3). The experimental surface temperature measurements match well with the computations (SI Fig. S2). The numerical results reveal that, at steady-state, the minimum temperature occurs at the surface while the maximum temperature occurring at the heaters is within 5 ^∘^C, thereby confirming that the interior temperatures are below *T*_*g*_. The experimental transient in situ heating and cooling behavior of GFRP and CFRP composites is depicted in Figure 3f, as obtained from IR camera surface temperature measurements. Within 10 min, the target 130 ^∘^C healing temperature is reached on the top surface of the composites and maintained for an additional 5 min before 30 min of cooling to RT. This sub-hour healing protocol is over 50 times faster than state-of-the-art, in situ microvascular healing systems^18,43^.

### In situ self-healing

**Fig. 4:**
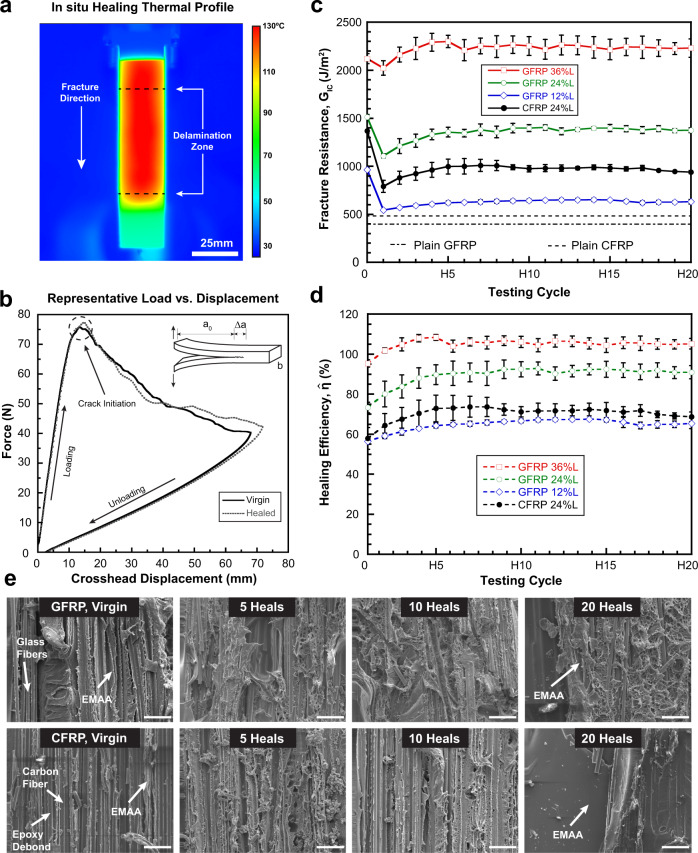
Mode-I fracture and in situ self-healing performance. **a** Top surface temperature distribution of a fractured double cantilever beam (DCB) during a self-healing thermal remending cycle. **b** Representative load versus displacement behavior for a virgin and self-healed DCB specimen. **c** Delamination resistance (i.e., *G*_IC_) evolution for GFRP and CFRP specimens at various as-printed EMAA areal coverages (12, 24, and 36%) over 20 heal cycles; dashed lines indicate values for plain (unmodified) composites. Error bars represent the standard deviation from the mean (*n* = 3). **d** Healing efficiency ($\hat{\eta }={G}_{{{{{{{{\rm{IC}}}}}}}}}^{{{{{{{{\rm{healed}}}}}}}}}/{G}_{{{{{{{{\rm{IC}}}}}}}}}^{{{{{{{{\rm{virgin}}}}}}}}}$) behavior for self-healing composite laminates in (**c**). Error bars represent the standard deviation from the mean (*n* = 3). **e** Scanning electron micrographs (SEM) of fracture surfaces in GFRP (top) and CFRP (bottom) composites after virgin, 5 heal, 10 heal, and 20 heal test cycles showing EMAA microporous network development and distribution evolution (scale bars = 50 μm).

Fig. 4a shows the surface temperature profile for a double cantilever beam (DCB) mode-I fracture specimen during in situ self-healing after delamination is propagated through the EMAA architectured mid-plane (see Methods Section). The DCB remains in the load frame during the entire thermal remending process. Representative force versus displacement behavior for a virgin and first post-repair test (i.e., heal cycle 1) are plotted in Fig. 4b. Typical linear-elastic response is followed by crack initiation and 50 mm of delamination propagation upon which the sample is unloaded to zero crosshead displacement. Self-healing commences via in situ thermal remending at *T*_*h*_ (≈130 ^∘^C) for 15 min with a 30 min cool down to RT, upon which the sample is reloaded/unloaded. Healing at even lower temperatures (i.e., 110 ^∘^C) is also demonstrated (SI Sec. 4). The area enclosed within the load-displacement curves represents the mode-I critical strain energy release rate (i.e., *G*_IC_), an established metric for quantification of fracture recovery^26^.

Figure 4c compares the healing performance of glass-fiber-reinforced polymer composite (GFRP) with varying as-printed areal coverages of EMAA in the mid-plane (12, 24, and 36%) over twenty consecutive heal cycles. In comparison to a plain (i.e., unmodified composite), the micro-patterned EMAA domains produce a pronounced (200–500%) increase in delamination resistance (*G*_IC_) that varies linearly with increasing areal coverage (SI Sec. 5). Self-healing performance over 20 cycles is characterized by an initial drop from virgin fracture resistance (albeit of the already toughened composite), with a gradual increase to relatively stable recovery. Control GFRP samples (with heaters but without EMAA) do not exhibit recovery under the same test conditions (SI Sec. 6). In situ self-healing is achieved in EMAA-patterned CFRP composites, without electrically shorting the heaters through the conductive carbon reinforcement, due to built-in dielectric glass-fiber layers insulating the resistive heaters. Virgin fracture resistance for the 24% areal coverage of EMAA in CFRP is slightly lower compared to an equivalent coverage in GFRP, but exhibits similar self-healing repeatability and consistency. *Healing efficiency*, calculated as the ratio of healed to virgin fracture resistance (i.e., $\hat{\eta }$ = ${G}_{{{{{{{{\rm{IC}}}}}}}}}^{{{{{{{{\rm{healed}}}}}}}}}/{G}_{{{{{{{{\rm{IC}}}}}}}}}^{{{{{{{{\rm{virgin}}}}}}}}}$), shows that over 100% recovery can be achieved with a 36% areal coverage in GFRP (Fig. 4d).

To further elucidate the fracture behavior and a new finding—GFRP exhibits greater healing efficiency than CFRP— topological investigation via scanning electron microscopy of the DCB fracture surface is conducted at different stages during mechanical testing (virgin and 5, 10, 20 heals). The delamination images in Fig. 4e reveal increased microporosity and distribution of EMAA across the fibrous fracture topology upon in situ thermal remending. This increase can be attributed to molten polymer flow assisted by self-pressurization from water vapor produced via the thermally-driven condensation reaction between the methacrylic acid group in EMAA and residual amines/hydroxyl groups in the epoxy matrix^46,47^. Cohesive failure through the toughened microporous network of EMAA produces a sufficient number of interfaces that regain contact after elastic unloading and dynamic bond reassociation, resulting in high (>100%) fracture recovery, i.e., delamination resistance. Despite steady improvement and eventual plateau in healing performance for both material platforms, the microporous EMAA network in glass-fiber composites is pervasive up to heal cycle 20. In contrast, carbon-fiber composites exhibit a smooth EMAA fracture topology by the final (20th) heal cycle.

**Fig. 5:**
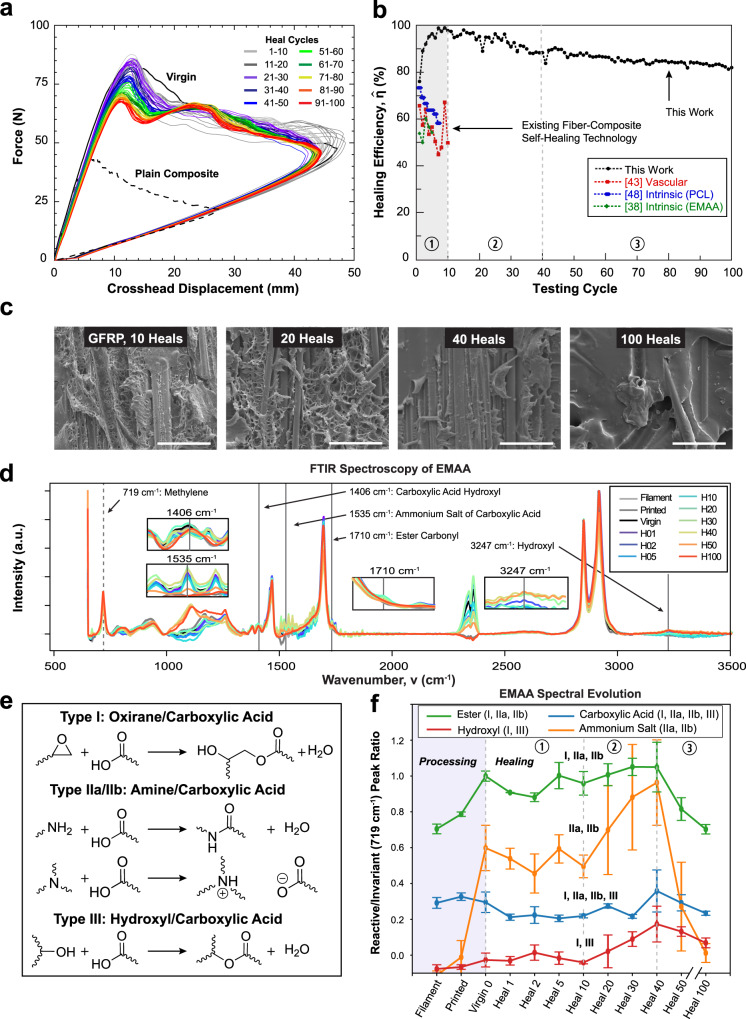
Prolonged self-healing demonstration and topological/spectroscopic evaluation. **a** Fracture behavior for a self-healing GFRP specimen (24% EMAA coverage) tested to 100 heal cycles. **b** Comparison of healing efficiency ($\hat{\eta }$) versus cycle count for the sample in (**a**) and other state-of-the-art self-healing composite technologies from the literature^38,43,48^. **c** Scanning electron micrographs (SEM) of the fractured GFRP specimen after 10, 20, 40, and 100 heals (scale bars = 50 μm). **d** Overlaid Fourier-transform infrared (FTIR) spectra of EMAA at varying thermal processing and mechanical testing steps with important peaks corresponding to active (solid vertical line) and invariant (dashed line) chemical groups. **e** Four key chemical reactions between EMAA and species present in the composite laminate are capable of covalent/ionic bonding. **f** Normalized active (1406, 1535, 1710, 3247 cm^−1^) to invariant (719 cm^−1^) peak ratios as a function of thermal processing and mechanical testing. Error bars represent the standard deviation from the mean (*n* = 3).

Continued testing of a GFRP specimen (24% EMAA areal coverage) reaches 100 heal cycles (Fig. 5a)—an order of magnitude greater than leading studies^38,43,48^. The recovery exhibits a modest decrease from a peak — $\hat{\eta }\approx 100\%$ in heal cycle 10 — to a value of $\hat{\eta }\approx 82\%$ by heal cycle 100 (Fig. 5b). The more stable mechanical behavior in GFRP for 40+ cycles corresponds to the progressive collapse of the microporous EMAA network, as revealed by scanning electron microscopy (Fig. 5c). The smooth fracture topology observed earlier in CFRP by heal cycle 20 is mirrored much later (by heal cycle 100) in the glass-fiber composite. The evolution of healing performance is attributed to the interplay of chemical and physical processes: flow of molten EMAA assisted by the formation of micropores, irreversible covalent and ionic bonding to composite constituents (fiber, matrix), and reversible hydrogen bonding between cohesively fractured EMAA surfaces.

### Chemical mechanisms

Prior studies describing the chemical reactions responsible for EMAA microporous network formation have been conducted solely on neat epoxy or carbon-fiber-reinforced composites^46,47^. Thus, to reveal if the same mechanisms are also responsible for the topological differences in glass-fiber composites, we conducted Fourier-transform infrared (FTIR) spectroscopy with attenuated total reflectance (ATR) for surface interrogation. ATR-FTIR spectra are acquired from neat EMAA at successive melt-processing steps and also from EMAA on virgin and repeatedly healed DCB fracture surfaces (Fig. 5d) to capture the full progression of relevant chemical groups. Four chemical reactions^46,47^—which rely on covalent and ionic bonding between EMAA and the matrix epoxide (i.e., oxirane), matrix amine, and surrounding hydroxyl groups—are examined in detail (Fig. 5e). Molecular vibrations corresponding to key chemical species (1406 cm^−1^ carboxylic acid hydroxyl stretch, 1535 cm^−1^ carboxylic acid ammonium salt stretch, 1710 cm^−1^ ester carbonyl stretch, and 3247 cm^−1^ hydroxyl stretch) are normalized to invariant methylene rocking peak at 719 cm^−1^, summarized in Fig. 5f. Assimilating these spectral signatures with scanning electron micrographs (SEM) of fracture surfaces, the entire GFRP healing profile (Fig. 5b) can be explained in terms of three primary zones. In the first zone (<10 cycles), the rapid increase in healing performance is primarily attributed to the spreading of molten EMAA promoted by repeated cycling and microporosity formation (Fig. 5c) resulting from the condensation reactions that produce water vapor. Healing efficiency increases linearly with EMAA areal coverage (SI Fig. S4), where microporosity enhances crack tortuosity, thereby increasing fracture resistance/recovery^49^. Once EMAA spreading has subsided, the second zone (>10 and <40 cycles) is characterized by a decrease in healing due to waning microporosity. This reduction in fracture recovery (<10%) is mitigated by covalent bonding between EMAA and composite interfaces, evidenced by increasing spectral intensities of amine, ester, and hydroxyl groups (Fig. 5f). Beyond heal cycle 40—the third zone—less than 5% drop in healing efficiency occurs over an additional 60 cycles. We accredit this stabilized recovery to reversible hydrogen bonding in cohesively fractured EMAA, revealed by a reduction in the measured peak intensities of covalent and ionic chemical groups and the eventual absence of microporosity in respective SEM. Similar spectral trends were observed in CFRP, however, occurring within a smaller window of 20 heal cycles (SI Sec. 7). We attribute this latest discovery—of increased healing performance in GFRP over CFRP (for an equivalent EMAA coverage)—to additional hydroxyl groups from the E-glass reinforcement and typical silane coating (i.e., sizing) applied to glass fibers for enhanced matrix bonding^50^. More significant is the scientific deduction that prolonged healing in both composites (SI Sec. 8) is primarily due to cohesive and reversible hydrogen bond reassociation via thermal remending of EMAA: a key evidence that the newly developed in situ self-healing system possesses the capacity to be perpetual. Needless to say, the recovery behavior beyond 100 healing cycles—specifically how much longer the healing will sustain—is yet to be determined. However, our results do not exhibit any signatures suggesting the healing will wane for any immediate cycles.

Harnessing this newfound synthetic material platform that embodies nature’s innate capacity for self-repair provides a promising pathway for sustainable infrastructure. Creating lightweight, resilient fiber composites—that minimize economic and environmental impacts—will offset thermoset recycling challenges and energy demands^9,51^. In addition to mechanical advantage, our micro-architectured composites enable multifunctionality^52^: for example, integrated heaters provide a green alternative to adverse chemicals used for deicing grounded aircraft and also engender aerosurfaces with anti-icing capacity during flight. Notably, one can realize our materials system using existing fiber-composite fabrication and scalable additive manufacturing with commodity EMAA copolymers. Thus, translating this laboratory research into industrial technology can be streamlined. Several aspects warrant future investigation, including (i) how external stimuli such as humidity, freeze/thaw cycles, and fatigue loading affect performance and (ii) developing mathematical models of thermal remending to provide deeper insight into the underlying mechanics. Nevertheless, after decades of global scientific iteration since self-healing inception^14^, we have developed and demonstrated prolonged self-healing in fiber composites without compromising structural integrity during and after in situ repair.

## Methods

### Materials

The same epoxy resin/amine hardener system (Araldite/Aradur 8605, Huntsman Advanced Materials, LLC) is used for all fabricated composites. For GFRP specimens, the E-glass reinforcement is Style 7781 8-harness (8H) satin weave fabric (Fibreglast) with an areal density of 305 gm^−2^. For CFRP specimens, the PAN-based carbon-fiber reinforcement is Style 94407 8H satin weave fabric (BGF Industries) with an areal density of 370 gm^−2^. Poly(ethylene-co-methacrylic acid), EMAA, is purchased in pelletized form (Nucrel^TM^ 2940, DuPont, Inc.). Resistive heating elements are non-perforated continuous textiles (PowerFilm™ EFG01-F0-0330-150-C02, LaminaHeat, LLC). Internal electrical connections to the resistive heater bus bars are made using copper tape with conductive adhesive (Product 1181, 3M, Inc.), silver paint (Product 16040-30, Ted Pella, Inc.), and copper wire (0.81 mm diameter). External electrical connections to the fabricated composites are made using copper wire (0.64 mm diameter) and conductive silver epoxy (Product 8331, MG Chemicals, Inc.) for GFRP specimens and dielectric epoxy (Gorilla Clear Epoxy, Maine Wood Concepts, Inc.) for CFRP specimens. Structural adhesive (Scotch-Weld DP460NS, 3M, Inc.) is used for securing the copper wire to resistive heater bus bars, steel hinges (25 mm × 25 mm, McMaster-Carr, Inc.) to composite DCB specimens, and fiberglass tabs (Garolite G-10/FR4, Mcmaster-Carr, Inc.) to composite tensile specimens.

### EMAA filament-extrusion

EMAA filament (≈2.6 mm diameter) for 3D printing is produced using a single-screw extruder (Filastruder, LLC) with a 3 mm diameter circular die and a barrel temperature of 110 ^∘^C with a room temperature (RT) take-up reel (≈170 mm diameter) rotating at two revolutions per minute (RPM).

### 3D printing EMAA on fiber-reinforcement

Extruded EMAA filament is printed directly on glass- and carbon-fiber reinforcement (254 mm × 254 mm) placed atop the heated build plate of a fused-deposition modeling (FDM) machine (TAZ 5, Lulzbot, Inc.). During printing through a 0.35 mm nozzle, the hot end and build plate temperatures are maintained at 170 and 60 ^∘^C, respectively. EMAA is printed in serpentine patterns with the primary traces oriented parallel to the longitudinal axis of test specimens. Areal coverages of 12, 24, and 36% correspond to center-to-center trace spacings of 4.90, 2.23, and 1.44 mm, respectively. As-printed cross-sections are elliptical (≈500 μm wide × 310 μm tall).

### Internal electrical connections to resistive heaters

For the self-healing specimens, resistive heating textile (254 × 140 mm, width × length) is marked along both continuous rows of bus bars to delineate centerline locations of eight (25 mm wide) samples. A steel razor blade is used to perforate the top and bottom layer of E-glass fabric encasing the bus bars, making a 15 mm slit from the outer edges along the sample centerlines. The perforations are coated with conductive silver paint, which dries for 30 min before placing a 25 mm long segment of copper wire (0.81 mm diameter) in each slit (i.e., 10 mm overhang) and overcoating with silver paint to ensure a sound electrical connection. After an additional 30 min of drying, the connection is potted with a thin layer of structural adhesive (DP460NS), secured using a single layer of conductive copper tape, and cured at 49 ^∘^C for 4 h.

### Melt consolidation of EMAA in composite preforms

Glass- and carbon-fiber reinforcing plies with 3D printed EMAA patterns are placed at the mid-plane of a respective stacking sequence forming a composite preform (see subsequent specimen fabrication sections for layup details). Fully-constructed composite preforms (254 × 254 mm) are placed between a pair of aluminum plates (406 × 406 × 6.35 mm) and weighted to a total pressure of 1 kPa relative to the surface area of the composite preform. The entire preform/plate assembly is placed into a mechanical convection oven (StableTemp EW-52412-79, Cole-Parmer, Inc.) and heated from room temperature to 110 ^∘^C over a 15 min period. The temperature is held at 110 ^∘^C for 75 min and then cooled to 60 ^∘^C over 90 min prior to removal from the oven. The weighted assembly is then allowed to cool to room temperature (RT ≈ 23 ^∘^C) prior to extraction of the melt-consolidated preform.

### Vacuum-assisted resin transfer molding (VARTM)

Liquid epoxy resin/amine hardener infiltration (Araldite/Aradur 8605, 100:35 by wt.) is achieved using VARTM at 2 Torr (abs) until complete fabric wetting and then decreased to 380 Torr (abs) for 24 h at RT until matrix solidification. The infused composite panels are cured for 2 h at 121 ^∘^C followed by 2 h at 150 ^∘^C to yield a final glass-transition temperature (*T*_*g*_ ≈140 ^∘^C) as measured by dynamic mechanical analysis (see the subsequent section for details on thermo-mechanical characterization).

### External electrical connections for self-healing specimens

Post-composite fabrication, samples are cut to size (25 mm wide × 140 mm long) from the ≈4 mm thick panel using a diamond-blade wet saw, exposing the four embedded copper wire (0.81 mm diameter) cross-sections at each end. A 0.65 mm diameter center hole is drilled 4 mm deep into each embedded wire, whereby copper wire (0.64 mm diameter) coated with conductive silver epoxy is inserted into each hole and allowed to sit for 24 h at RT to develop sufficient adhesive bonding strength.

### Fabrication of uniaxial tension specimens

Plain composite textile preforms consist of sixteen layers of 2D woven plies in an alternating [0/90] sequence that is geometrically symmetric about the mid-plane, where 0 indicates the warp (7 tows) direction and 90 indicates the weft (1 tow) direction when viewed from above. GFRP contains 16 plies of Style 7781 8H satin woven fabric and CFRP contains eight plies of Style 94407 8H satin woven fabric. Additional sample types containing a printed EMAA ply at the mid-plane (type 1) or two resistive heater interlayers replacing two reinforcing plies (type 2) or a printed EMAA mid-plane ply plus two resistive heater interlayers (type 3) are constructed by selectively patterning EMAA. Preforms that produce plain and type 1 specimens have a stacking sequence of [90/0]_4_-EMAA-[90/0]_4_ for GFRP or [90/0]_2_-EMAA-[90/0]_2_ for CFRP, with EMAA serpentines printed atop the middle ply (for type 1) of every other sample. Preforms that produce type 2 and type 3 specimens have a stacking sequence of [0/90]_2_-heater-[0/90/0]-EMAA-[90/0/90]-heater-[0/90]_2_ for GFRP or [0/90]-heater-0-EMAA-90-heater-[0/90] for CFRP, with EMAA serpentines printed atop the middle ply (for type 3) of every other sample. All preforms are melt-consolidated prior to matrix incorporation via VARTM.

Composite samples are cut using a diamond-blade wet saw from the ≈4 mm thick panels to 20 mm wide × 254 mm long for GFRP and 20 mm wide × 254 mm long for CFRP. To prevent sample damage in the load-frame grips during tension testing, fiberglass tabs (identical width, 45 mm long) are bonded to each face with structural adhesive cured at RT for 24 h plus 4 h at 49 ^∘^C to develop full strength. The tension samples are prepared for digital image correlation (DIC), by spray painting the front, back, and side profiles matte white followed by speckling with matte black paint to produce a high-contrast pattern.

### Fabrication of dynamic mechanical analysis (DMA) specimens

Neat epoxy samples (≈2.1 mm thick) are produced via cell casting between glass plates with a rubber gasket and cured for 24 h at RT until resin solidification, followed by 2 h at 121 ^∘^C, and 2 h at 150 ^∘^C. Plain composite samples (≈1.85 mm thick) contain 2D woven plies in an alternating sequence, where GFRP consists of eight plies [0/90]_4_ and CFRP consists of four plies [0/90]_2_. Preforms are infused via the VARTM process and cured at the same conditions as the cell castings. Both the neat epoxy and plain composite samples are cut to 10 mm wide and 60 mm long using a diamond-blade wet saw.

### Fabrication of fracture specimens

All composite preforms are geometrically symmetric about the mid-plane, whereas plain composites consist only of primary (glass/carbon) reinforcing plies and self-healing samples contain two resistive heater layers (with wired electrical connections) replacing respective reinforcement plies and a middle ply patterned with EMAA. Due to differing structural fabrics, plain GFRP contains 16 (self-healing: 14) plies of Style 7781 8H satin weave and plain CFRP contains eight (self-healing: six) plies of Style 94407 8H satin weave. The assembled preforms have the following stacking sequences: GFRP — plain [0/90]_8_, self-healing—[0/90]_2_-heater-[0/90/0]-EMAA-[90/0/90]-heater-[0/90]_2_ and CFRP—plain [0/90]_4_, self-healing — [0/90]-heater-0-EMAA-90-heater-[0/90]. A 25 μm thick ethylene tetrafluoroethylene (ETFE) film (full panel width and 50 mm long) is also placed atop the mid-plane layer to serve as a pre-crack. All self-healing preforms are melt-consolidated and then infused via the VARTM process, followed by curing. Samples are then cut using a diamond-blade wet saw from the ≈4 mm thick panel to 25 mm wide × 140 mm long. Steel hinges are bonded to outer composite faces on the pre-crack end with structural adhesive and cured at room temperature for 24 h plus 4 h at 49 ^∘^C to reach full strength. For self-healing samples, the top face is spray-painted matte black for infrared imaging and external electrical connections are made as previously described. A line 50 mm from the interior edge of the pre-crack is delineated on the bottom and side faces to mark the termination point for delamination propagation during fracture testing.

### Fracture testing and self-healing evaluation

Mode-I fracture testing of double cantilever beam (DCB) specimens is conducted in accordance with ASTM D5528 using a 10 kN electromechanical load frame (Alliance RT/5, MTS, Inc.) equipped with a 250 N load cell. The initial pre-crack region (*a*_0_) from the hinge-loading line to the interior ETFE film termination interface is ≈35 mm. Samples are loaded to an incremental crack length (Δa) of 50 mm at 5 ${{{{{{{\rm{mm}}}}}}}}\,{\min }^{-1}$ and subsequently unloaded at the same rate to the initial position (i.e., zero crosshead displacements) for all test cycles. A high-resolution digital camera (Rebel T7, Canon, Inc.) with a macro lens is mounted underneath the specimen (for GFRP composites) or in profile with the specimen (for CFRP composites) in conjunction with appropriate lighting to accurately monitor crack front propagation.

In situ self-healing via thermal remending is performed at zero crosshead displacement within the load frame. Resistive heating commences via electrical power application of ≈12 W for GFRP and ≈13.5 W for CFRP to the textile resistive reinforcement layers using a DC power supply (Model 4602, Tektronix, Inc.). Power is supplied for 15 min to achieve a maximum top surface temperature of 130 ^∘^C, as monitored by an overhead infrared camera (FLIR A600, Teledyne FLIR, Inc.) before being disconnected and allowing the specimen to cool for 30 min to RT. Specimens are then reloaded to assess self-healing efficiency via crack-growth resistance.

For all testing cycles, mode-I strain energy release rate (*G*_I_)—a measure of crack-growth resistance—is calculated using the area method^53^ according to the expression:

1 $${G}_{{{{{{{{\rm{I}}}}}}}}}=\frac{1}{b}\frac{\Delta U}{\Delta a},$$

where *b* is the sample width, Δ*a* is the measured change in crack length, and Δ*U* is the change in internal work or strain energy due to elastic bending in the cantilever arms. Mathematically, Δ*U* can be derived from energy principles as the area under the load-displacement curve at an incremental crack length (Δa):

2 $$\Delta U=\int\limits_{0}^{\hat{\delta }}Pd\hat{\delta }{|}_{\Delta a},$$

where *P* represents the measured force at a prescribed crosshead displacement $\hat{\delta }$. (To avoid conflict in notation with $\tan \delta$, employed in the thermo-mechanical analysis discussed below, we have used $\hat{\delta }$ to denote the displacement.) Healing efficiency is calculated as the ratio between healed and virgin critical strain energy release rates according to the established relation^26^:

3 $$\hat{\eta }:=\frac{{G}_{{{{{{{{\rm{IC}}}}}}}}}^{{{{{{{{\rm{healed}}}}}}}}}}{{G}_{{{{{{{{\rm{IC}}}}}}}}}^{{{{{{{{\rm{virgin}}}}}}}}}}\times 100,$$

where $\hat{\eta }$ is the healing efficiency expressed in percentage, and ${G}_{{{{{{{{\rm{IC}}}}}}}}}^{{{{{{{{\rm{virgin}}}}}}}}}$ and ${G}_{{{{{{{{\rm{IC}}}}}}}}}^{{{{{{{{\rm{healed}}}}}}}}}$ are the virgin and healed critical strain energy release rates, respectively.

### Thermo-mechanical testing

Dynamic mechanical analysis (DMA) is performed on neat epoxy and plain composite samples in accordance with ASTM E1640. A dynamic mechanical analyzer (Q800, TA Instruments, Inc.) with a 3-pt bending fixture (50 mm span) applies an oscillating strain of 0.1% at 1 Hz frequency following an initial preload of 0.01 N. A temperature sweep from RT to 250 ^∘^C is conducted at a ramp rate of ${5}^{\circ }{{{{{{{\rm{C}}}}}}}}\,{\min }^{-1}$ while storage modulus (${{{{{{{{\rm{E}}}}}}}}}^{\prime}$), loss modulus (E^*″*^), and tan *δ* data were collected at a sampling rate of 0.5 Hz. The peak of tan *δ* is recorded as the glass-transition temperature (*T*_*g*_) for each sample.

### Uniaxial tension testing

Tabbed tension samples are aligned and clamped in mechanical wedge grips and quasi-statically tested in accordance with ASTM D3039. A crosshead displacement rate of 1.5 ${{{{{{{\rm{mm}}}}}}}}\,{\min }^{-1}$ is applied using a 100 kN load frame (Exceed E45, MTS, Inc.) equipped with a 100 kN load cell. Concurrent images for DIC are acquired using two 12.3 MP video cameras (GS-3-U3-15S5C-C, Teledyne FLIR, Inc.) to capture the full-field displacements of the front and back sample faces. Time-stamped images are analyzed using image processing software (VIC-2D, Correlated Solutions, Inc.) to calculate respective strain components.

### Optical microscopy

Images of polished composite cross-sections are acquired using an optical microscope (AXIO Zoom.V16, Zeiss, Inc.) equipped with a coaxial light source and polarization filter. Image compilations are created from tiled individual images that are acquired with a motorized stage and automatically stitched using software control (Zen 2.3 Pro, Zeiss, Inc.).

### Scanning electron microscopy

Scanning electron micrographs (SEM) are acquired using a variable pressure scanning electron microscope (3200N, Hitachi, Ltd.) at a 10 kV accelerating voltage after sputtering the samples with gold/palladium to a target coating thickness of 50 nm.

### X-ray computed microtomography

Data is acquired using a high-resolution 3D X-ray imaging system (Xradia 510 Versa, Zeiss, Inc.). 360^∘^ scans are collected in rotation intervals of 0.225^∘^ using a 0.4x objective at 10 s exposure times with a 160 kV (10 W, 62.5 μA) source. 3D reconstructions are created from 1600 projections using onboard software, and visualizations are produced using third-party software (Dragonfly v2021.3, Object Research Systems, Inc.)

### Fourier-transform infrared (FTIR) spectroscopy

Spectroscopy is performed on post-fractured DCB sections using an FTIR microscope (HYPERION 1000, Bruker, Inc.) in attenuated total reflectance (ATR) mode with a 70 μm germanium crystal probe and 15x objective. Thirty-two scans are collected at each sampling site from the 4000 to 650 cm^−1^ wavenumber range.

## Supplementary information

Supplementary Information

## Data Availability

The authors declare that the main data supporting the findings of this study are available within the article and the Supplementary Information files. Source data files are available from the corresponding author upon request and with approval from NC State University.
