# Supplementary material for: Prolonged in situ self-healing in structural composites via thermo-reversible entanglement
Source: Nat Commun. 2022 Oct 31;13:6511. doi: 10.1038/s41467-022-33936-z (PMC9622832; doi:10.1038/s41467-022-33936-z)
Supplement: Supplementary file 1 — Supplementary Information [file 41467_2022_33936_MOESM1_ESM.pdf]

Supplementary Information

Prolonged in situ Self-healing in Structural Composites via Thermo-reversible Entanglement

Alexander D. Snyder[1], Zachary J. Phillips[2], Jack S. Turicek[1], Charles E. Diesendruck[3], Kalyana B. Nakshatrala[4], & Jason F. Patrick[1,2*]

[1]Department of Mechanical and Aerospace Engineering, North Carolina State University

[2]Department of Civil, Construction, and Environmental Engineering, North Carolina State University

[3]Schulich Faculty of Chemistry, Technion–Israel Institute of Technology

[4]Department of Civil and Environmental Engineering, University of Houston

*Corresponding author, e-mail: jfpatric@ncsu.edu

Supplementary Note 1. Differential scanning calorimetry (DSC) and melt rheology of EMAA

Dynamic mechanical analysis (DMA) is performed at varying temperatures according to the procedure outlined in the respective Experimental Section: "Thermo-mechanical testing". The peak of tan δ is recorded as the glass-transition temperature (T_g) for each sample.

Table S1: Neat epoxy matrix and plain composite thermomechanical properties.

Sample type	T_g (°C)	Storage modulus (E') in GPa at various temperatures (% of the value at RT)					
		RT	T_g	$T_g + 10\,°C$	$T_g + 5\,°C$	$T_g - 5\,°C$	$T_g - 10\,°C$
**Neat epoxy matrix**	153.53	2.90	0.13 (4.48%)	0.05 (1.72%)	0.07 (2.48%)	0.28 (9.69%)	0.55 (20%)
**Plain GFRP**†	141.02	16.68	8.85 (53.05%)	5.95 (35.67%)	7.03 (42.15%)	11.15 (66.85%)	13.87 (83.15%)
**Plain CFRP**‡	140.48	25.70	13.50 (52.53%)	8.93 (34.74%)	10.60 (41.25%)	17.90 (69.65%)	22.20 (86.38%)

† Fiber volume fraction (V_f) for GFRP calculated as 51.3% per ASTM D3171 - Procedure G[1].

‡ Fiber volume fraction (V_f) for CFRP calculated as 54.1% per ASTM D3171 - Procedure B[1].

Supplementary Note 2. Differential scanning calorimetry (DSC) and melt rheology of EMAA

Differential scanning calorimetry (DSC) is performed on samples from extruded EMAA filament contained in hermetically sealed aluminum pans with a heat-flux DSC (Q2000, TA Instruments, Inc.). Samples are cooled to $0\,^{\circ}\mathrm{C}$ and held for 5 min before being heated to $300\,^{\circ}\mathrm{C}$ at $10\,^{\circ}\mathrm{C/min}$, and then cooled to $25\,^{\circ}\mathrm{C}$ at a rate of $10\,^{\circ}\mathrm{C/min}$ to complete a full heat-cool cycle. Each sample is successively cycled twice (I/II), with representative data provided in Fig. S1a. In the first heating cycle, an order-disorder transition occurs at $39.7\,^{\circ}\mathrm{C}$ due to reorientation of aggregates formed via secondary bonding between methacrylic acid moieties[2,3]. The endothermic peak observed at $85.9\,^{\circ}\mathrm{C}$ is attributed to the melting of crystalline polyethylene domains, whereas upon cooling, exothermic recrystallization occurs around $58.1\,^{\circ}\mathrm{C}$. Upon reheating, the translation of the order-disorder transition to a higher temperature ($46.9\,^{\circ}\mathrm{C}$) indicates that underlying relaxation mechanisms in the aggregate regions may occur at timescales exceeding the period between successive heat-cool cycles. The respective melting and recrystallization temperatures remain the same in the first and second thermal cycles, indicating that crystalline domain formation is primarily driven by cooling kinetics and mechanisms separate from those driving aggregate reorientation[2].

Parallel plate rheometry is performed on EMAA pellets (Nucrel™ 2940) using a hybrid rheometer (HR-1, TA Instruments, Inc.). The pellets are placed on the bottom platen at RT and held in place using a guide ring while being heated to an initial temperature of $100\,^{\circ}\mathrm{C}$ and melted into a monolithic film. The guide ring is then removed and the top platen is brought into contact with the film while preserving a gap of 1 mm relative to the bottom platen. A shear rate of $\dot{\gamma}$ = 0.1 s^{-1} is applied while the temperature (above all melt transitions) is ramped from $100\,^{\circ}\mathrm{C}$ to $200\,^{\circ}\mathrm{C}$ at $1\,^{\circ}\mathrm{C/min}$. Viscosity data as-collected is provided in Fig. S1b. As the temperature increases, viscosity continually decreases. At the lowest *in situ* healing temperature ($110\,^{\circ}\mathrm{C}$) the melt viscosity is nearly three times higher than the viscosity (440 Pa·s) at the highest healing temperature ($130\,^{\circ}\mathrm{C}$) studied, and roughly six times higher than at T_g ($\approx 140\,^{\circ}\mathrm{C}$).

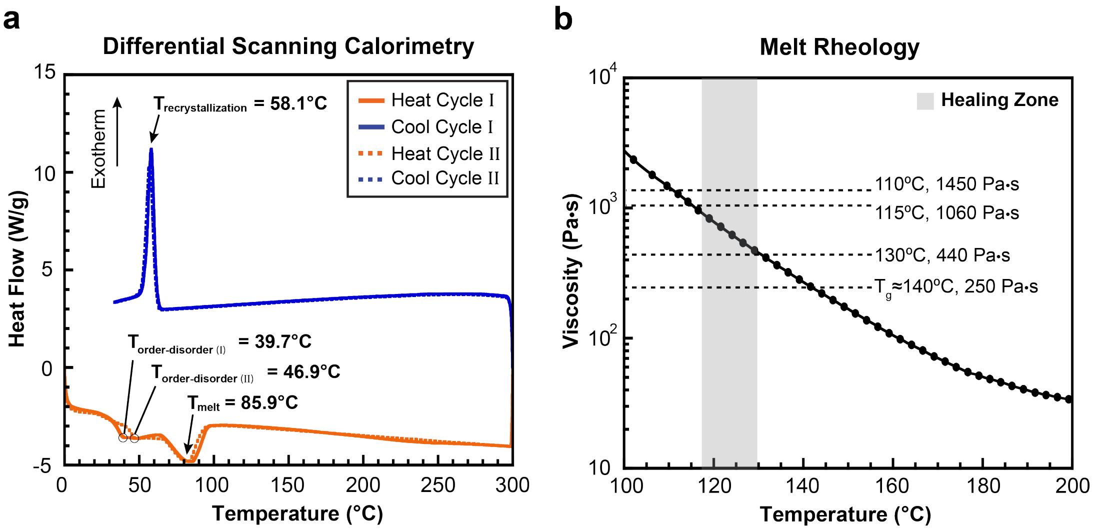

Fig. S1 Differential scanning calorimetry (DSC) and melt rheology of EMAA. a) Two successive DSC heat-cool cycles (I/II) revealing an order-disorder transition and single endothermic melting peak upon heating, and single exothermic crystallization peak upon cooling. b) Temperature-dependent melt viscosity of EMAA (Nucrel^{TM} 2940) measured by parallel-plate rheometry.

Supplementary Note 3. Three-dimensional heat transfer modeling

3D finite element heat transfer simulations are performed using weak form capability in COMSOL (version 5.6). The heat transfer model considers heat conduction within the bulk of the solid, and the boundary surfaces are free: both convection and radiation allowed. The embedded heaters serve as heat sources, modeled as thin surfaces. A Fourier model is used for conduction while Newton's law of cooling and Stefan-Boltzmann's law are, respectively, used for the convection and radiation components of heat transfer. The domain Ω is a cuboid representing a pristine DCB sample: the in-plane dimensions are given by the rectangle $L \times B$, and the out-of-plane dimension is the sample thickness d. The outer boundary surfaces are denoted by $\partial\Omega$. Two heaters are embedded running parallel to the in-plane and symmetrically placed with offset from the top and bottom surfaces that is dimensionally accurate to fabricated DCB composites.

The Galerkin weak formulation drives the finite element simulation; four-node tetrahedron elements with quadratic shape functions are employed. The Galerkin weak formulation reads:

$$\int_{\Omega} \mathrm{grad}[w] \cdot \mathbf{K}\, \mathrm{grad}[\vartheta]\, \mathrm{d}\Omega = \int_{\Sigma} w(\mathbf{x}) f_0\, \mathrm{d}\Gamma - \int_{\partial\Omega} w(\mathbf{x}) h_T (\vartheta(\mathbf{x}) - \vartheta_{\mathrm{amb}})\, \mathrm{d}\Gamma - \int_{\partial\Omega} w(\mathbf{x})\, \epsilon\, \sigma\, (\vartheta^4(\mathbf{x}) - \vartheta^4_{\mathrm{amb}})\, \mathrm{d}\Gamma \qquad \text{(S1)}$$

where $\vartheta(\mathbf{x})$ is the temperature field, $w(\mathbf{x})$ is the weighting function, Σ is the location of embedded heaters, $\mathrm{div}[\cdot]$ and $\mathrm{grad}[\cdot]$ are spatial divergence and gradient operators, ϑ_{amb} is the ambient temperature, h_T is the heat transfer coefficient, $\sigma = 5.67 \times 10^{-8}$ [$\mathrm{W/m^2/K^4}$] is the Stefan-Boltzmann constant, ϵ is the emissivity, and f_0 is the power supplied by the heater. The thermal conductivity takes the following anisotropic form:

$$\mathbf{K} = \begin{bmatrix} k_{11} & 0 & 0 \\ 0 & k_{22} & 0 \\ 0 & 0 & k_{33} \end{bmatrix}, \qquad \text{(S2)}$$

where k_{11} and k_{22} are the in-plane conductivities, and k_{33} is the out-of-plane conductivity.

Table S2: Parameters used in the numerical heat transfer simulations.

Parameter	Value for CFRP	Value for GFRP
Length L [mm]	140	140
Width B [mm]	25	25
Thickness d [mm]	4.15	4.25
Emissivity ϵ	0.95	0.95
Heat transfer coefficient h_T [$\mathrm{W/m^2/K}$]	13.125	13.23
Applied heater flux f_0 [$\mathrm{W/m^2}$]	2700	2400
Top/bottom surface offset for heaters [mm]	1.16	0.765
Ambient temperature ϑ_{amb} [°C]	23	23
Thermal conductivity (k_{11}, k_{22}, k_{33}) [$\mathrm{W/K}$]	(2.5247, 2.5247, 0.6019)	(0.5593, 0.5593, 0.3967)

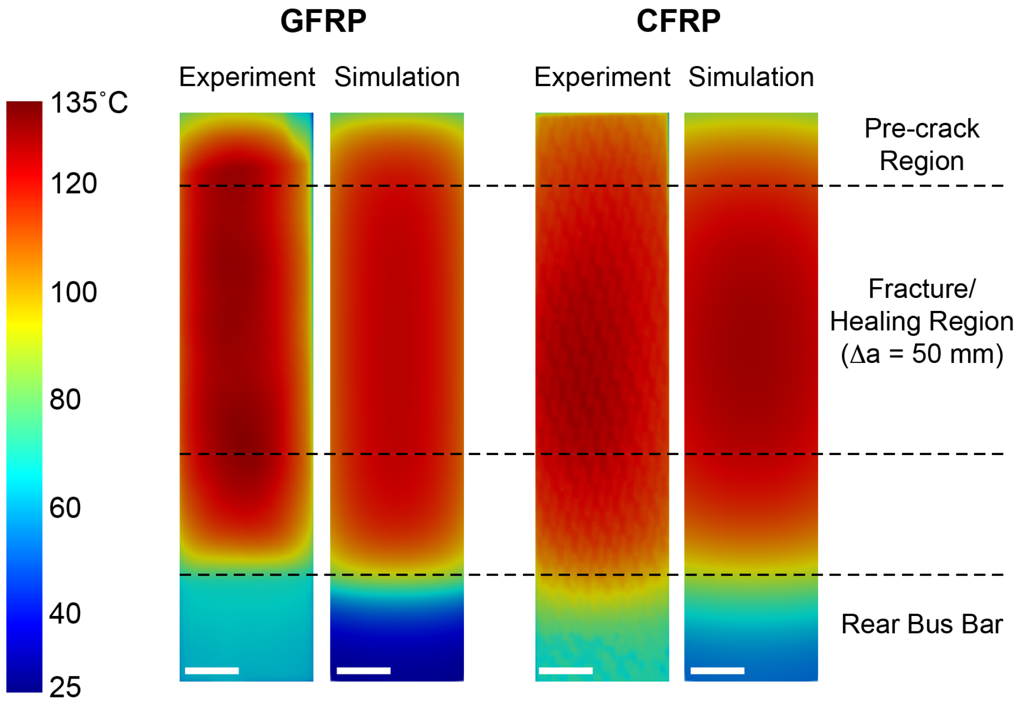

Fig. S2 Heat transfer investigation. Side-by-side comparison of experimentally measured and simulated top surface temperature contours for both GFRP and CFRP composite DCB specimens. Respective regions of interest are labeled, with the anterior region truncated in the computational results to match the IR camera field of view from experiments (scale bars = 10 mm).

Supplementary Note 4. Effect of thermal-remending temperature on *in situ* self-healing in GFRP

The effect of reducing the target healing temperature (110 °C) versus the standard target healing temperature of T_h = 130 °C is examined in GFRP composite specimens containing a midplane EMAA serpentine pattern with 24% as-printed areal coverage. As shown in Fig. S3, the 20 °C reduction in healing temperature results in lower mode-I fracture resistance (G_{IC}) across the virgin and all 20 heal cycles, but convergent healing efficiency ($\hat{\eta}$) of 90% by the final heal cycle. The results suggest self-healing may be influenced by melt-viscosity (440 Pa·s at 130 °C and 1450 Pa·s at 110 °C), though temperature-dependent kinetics of the associated chemical reactions could also play an important role in fracture recovery.

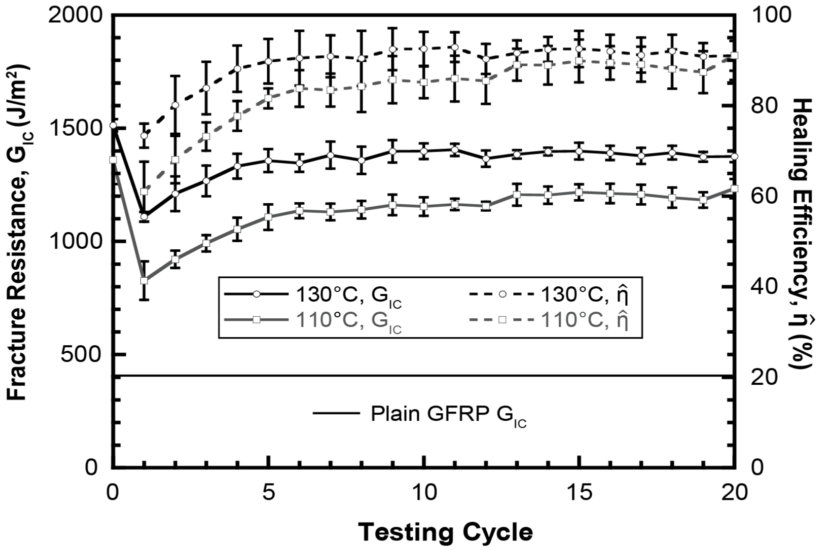

Fig. S3 Effect of healing temperature on GFRP *in situ* fracture recovery. Cyclic fracture resistance (G_{IC}) and healing efficiency ($\hat{\eta}$) behavior for self-healing GFRP composites at thermal-remending temperatures of 110 °C and 130 °C with 24% as-printed EMAA areal coverage. Error bars represent standard deviation from the mean (n = 3).

Supplementary Note 5. Effect of EMAA areal coverage on virgin delamination resistance

Fig. S4 shows the nearly linear increase in virgin mode-I fracture resistance (i.e., G_{IC}) with increasing as-printed areal coverage.

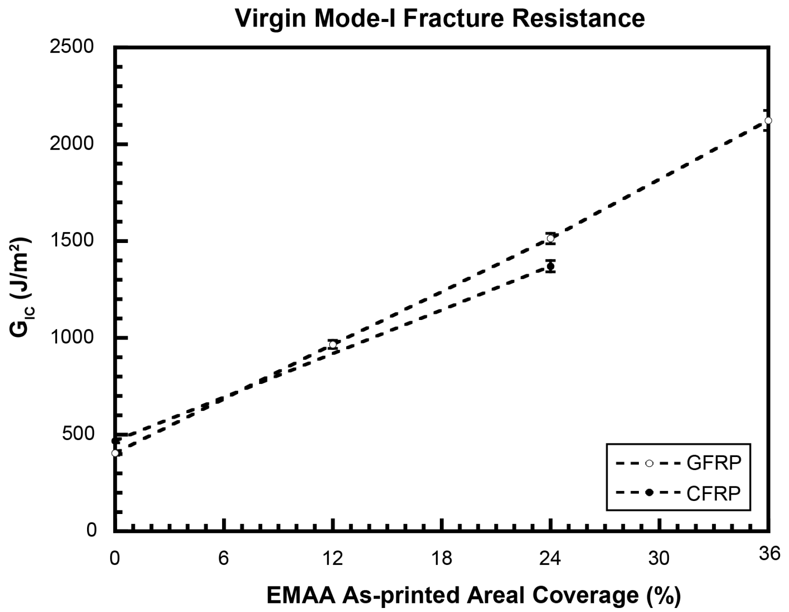

Fig. S4 Mode-I fracture resistance of EMAA-toughened composites. Relationship between as-printed areal coverage of EMAA midplane patterns and measured (virgin) interlaminar fracture resistance (G_{IC}) in GFRP and CFRP double cantilever beam (DCB) specimens. Error bars represent standard deviation from the mean (n = 3).

Supplementary Note 6. Control DCB study in GFRP without patterned EMAA

Fracture testing of GFRP double cantilever beam (DCB) control specimens— not containing EMAA but including resistive heaters and having a layup sequence of $[0/90]_3$-heater-[0/90]-heater-$[0/90]_3$— is conducted to confirm that even with *in situ* heating, no self-healing occurs in the absence of EMAA. Three specimens are manufactured identically to and subjected to the same mode-I loading and *in situ* heat/cool procedure as other self-healing GFRP composites detailed in the main manuscript. In Fig. S5 representative load versus displacement behavior for the virgin and

one subsequent test cycle (after *in situ* heating/cooling) shows negligible recovery of fracture resistance, evidenced by the virgin unload and subsequent loading paths having the same compliance (i.e., slope) resulting from identical crack lengths—thus, no appreciable fracture repair occurs.

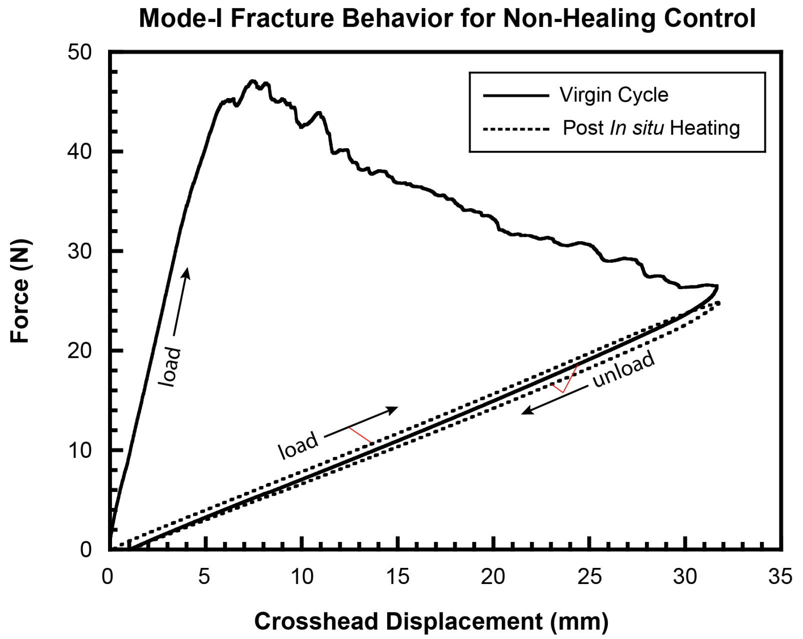

Fig. S5 Representative mode-I fracture behavior for non-healing GFRP control. Force versus displacement (load/unload) curves for virgin and one subsequent test cycle post *in situ* heating/cooling.

Supplementary Note 7. Fourier-transform infrared spectroscopy (FTIR) of EMAA in carbon-fiber composites

As described in detail within the main text, Fourier-transform infrared (FTIR) spectroscopy with attenuated total reflectance (ATR) is able to track chemical species involved in the reaction(s) of EMAA and the surrounding composite (Fig. S6a); studied here in CFRP as opposed to GFRP. In particular, four chemical (I, IIa, IIb, III) reactions (Fig. 5e) rely on covalent and ionic bonding between EMAA and the matrix epoxide (i.e, oxirane), matrix amine, and surrounding hydroxyl groups. Fig. S6b summarizes molecular vibrations corresponding to key active groups (1406 cm^{-1} carboxylic acid hydroxyl stretch, 1535 cm^{-1} carboxylic acid ammonium salt stretch, 1710 cm^{-1} ester carbonyl stretch, and 3247 cm^{-1} hydroxyl stretch) normalized to an invariant methylene

rocking peak at 719 cm^{-1}. Similar global trends to those in GFRP are observed (Fig. 5f), however, the reductions in peak intensity for the ester and ammonium salt occur much sooner in CFRP (heal cycle 5) than in GFRP (heal cycle 20).

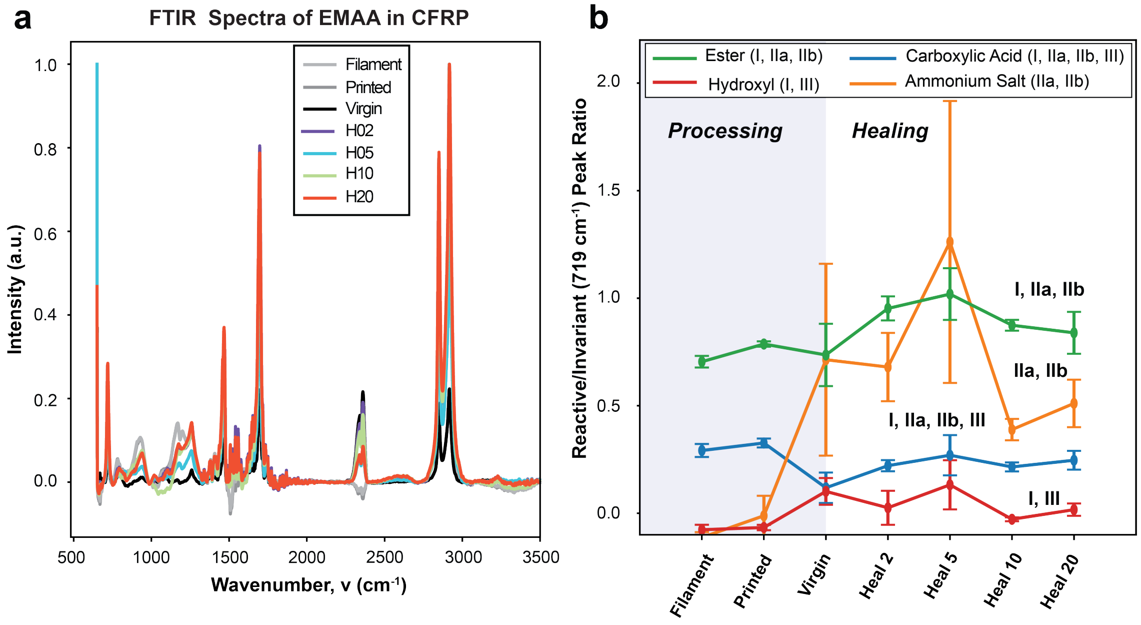

Fig. S6 Spectroscopic investigation of EMAA in self-healing CFRP composites. **a** FTIR spectra for EMAA at different processing steps and test cycles in CFRP composites. **b** Peak ratios of active species in the aforementioned chemical reactions (Fig. 5) to the invariant methylene rocking peak (719 cm^{-1}). Error bars represent standard deviation from the mean (n = 3).

Supplementary Note 8. Prolonged self-healing in carbon-fiber composites.

Fig. S7 shows prolonged self-healing performance in glass- and carbon-fiber composites where GFRP outperforms CFRP by a nearly constant difference in healing efficiency, with a mean (red line) and standard deviation of 23.7 $\pm$ 2.3% across all 100 heal cycles. Despite the aforementioned cessation of EMAA microporosity occurring sooner in CFRP ($\sim$20 cycles) than in GFRP ($\sim$40 cycles), self-healing in CFRP is also preserved and provides further evidence that cohesive, reversible hydrogen bonding is largely responsible for continued fracture recovery.

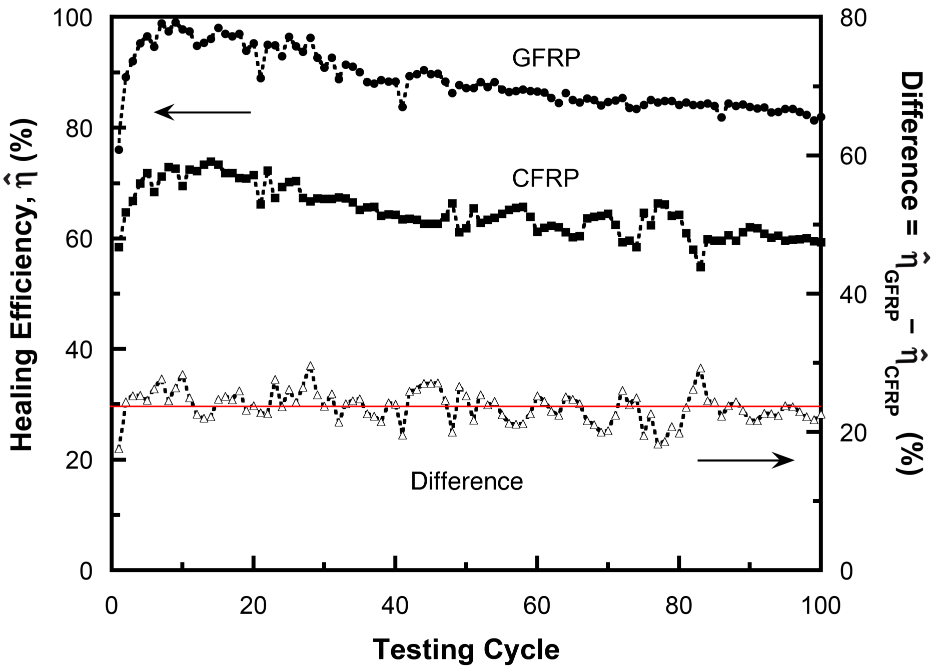

Fig. S7 Self-healing comparison of glass- (GFRP) and carbon-fiber (CFRP) composites. Healing efficiencies (left y-axis) for GFRP and CFRP samples along with the difference in healing (right y-axis) between composites over repeated test cycles. The red line represents the mean difference ($\approx$ 24%) across all 100 heal cycles.

Supplementary References

1. ASTM International. *ASTM D3171: Standard Test Methods for Constituent Content of Composite Materials* (2015).

2. Hirasawa, E., Yamamoto, Y., Tadano, K. & Yano, S. Formation of ionic crystallites and its effect on the modulus of ethylene ionomers. *Macromolecules* **22**, 2776–2780 (1989).

3. Kutsumizu, S. *et al.* Investigation of microphase separation and thermal properties of noncrystalline ethylene ionomers. 2. IR, DSC, and dielectric characterization. *Macromolecules* **33**, 9044–9053 (2000).
